# Kinematic and Neurophysiological Consequences of an Assisted-Force-Feedback Brain-Machine Interface Training: A Case Study

**DOI:** 10.3389/fneur.2013.00173

**Published:** 2013-11-07

**Authors:** Stefano Silvoni, Marianna Cavinato, Chiara Volpato, Giulia Cisotto, Clara Genna, Michela Agostini, Andrea Turolla, Ander Ramos-Murguialday, Francesco Piccione

**Affiliations:** ^1^Department of Neurophysiology, I.R.C.C.S. S.Camillo Hospital Foundation, Venice, Italy; ^2^Laboratory of Kinematics and Robotics, I.R.C.C.S. S.Camillo Hospital Foundation, Venice, Italy; ^3^Department of Information Engineering, University of Padua, Padua, Italy; ^4^Institute of Medical Psychology and Behavioral Neurobiology, Eberhard-Karls-University, Tübingen, Germany; ^5^TECNALIA, San Sebastian, Spain

**Keywords:** brain-machine interface, motor-training, proprioception, assisted-force-feedback, operant learning

## Abstract

In a proof-of-principle prototypical demonstration we describe a new type of brain-machine interface (BMI) paradigm for upper limb motor-training. The proposed technique allows a fast contingent and proportionally modulated stimulation of afferent proprioceptive and motor output neural pathways using operant learning. Continuous and immediate assisted-feedback of force proportional to rolandic rhythm oscillations during actual movements was employed and illustrated with a single case experiment. One hemiplegic patient was trained for 2 weeks coupling somatosensory brain oscillations with force-field control during a robot-mediated center-out motor-task whose execution approaches movements of everyday life. The robot facilitated actual movements adding a modulated force directed to the target, thus providing a non-delayed proprioceptive feedback. Neuro-electric, kinematic, and motor-behavioral measures were recorded in pre- and post-assessments without force assistance. Patient’s healthy arm was used as control since neither a placebo control was possible nor other control conditions. We observed a generalized and significant kinematic improvement in the affected arm and a spatial accuracy improvement in both arms, together with an increase and focalization of the somatosensory rhythm changes used to provide assisted-force-feedback. The interpretation of the neurophysiological and kinematic evidences reported here is strictly related to the repetition of the motor-task and the presence of the assisted-force-feedback. Results are described as systematic observations only, without firm conclusions about the effectiveness of the methodology. In this prototypical view, the design of appropriate control conditions is discussed. This study presents a novel operant-learning-based BMI-application for motor-training coupling brain oscillations and force feedback during an actual movement.

## Introduction

Motor rehabilitation in neurological disorders has been recently combined with neurophysiologic feedback of brain activity related to motor functioning to improve functional outcome. Based on observations of neural plasticity (1–4), assistive technologies, and robot-aided therapies are currently exploited (5) to augment visuospatial/proprioceptive feedback (6, 7) and provide an alternative rehabilitation strategy, especially in severe patients (8–11). These approaches include devices such as: robotic-arms, gait machines, treadmills, mechanical orthosis, and haptic devices suitable for finger, hand, and arm (5, 12). Other active rehabilitation treatments include: functional electrical stimulation (FES) devices (13) and virtual environments (6, 7). All these devices are usually employed in an open-loop modality, with a pre-defined or a user-adapted feedback according to assist-as-needed strategy (14, 15). Principles of a rewarding feedback during motor-training have been evaluated in many studies where reinforced or task-oriented feedback have been used with promising results in terms of functional recovery (5, 6, 12, 16–18). In one study a long-term robot-assisted treatment was compared with standard rehabilitation care showing a significant improvement of the Fugl-Meyer score (12). In another case the positive effects of a short-term goal-directed robotic therapy were sustained 4 months after the treatment (18). Other recent studies investigating the haptic-based intervention in an open-loop modality without brain activity proved its effectiveness in comparison to the standardized repetitive physiotherapy practice (19) and stability of the functional improvement (20). Because of the poor clinical outcome, these results seem to suggest that an enhanced brain-self-regulated proprioceptive assistance might be a promising modality for motor rehabilitation after stroke.

Recently, brain-machine interface (BMI) technology offers the possibility of brain self-regulation with immediate and reinforced feedback of motor-related brain activity. BMI provides a closed-loop, generally combining neurophysiological signals, an operant learning paradigm, and an external device (21–24). This combination allows the contingent stimulation of down-stream and afferent neural fibers to promote neural plasticity (2–4). Differently from the context considered in the present study, another way to promote brain plasticity relies on stimulation techniques operating directly on the central nervous system (3, 25).

Most BMI-studies on motor re-learning reported positive results (10, 11, 22, 26, 27) and are almost always characterized by the following main components: (i) the combination of BMI-training with goal-oriented physical practice, (ii) the involvement of severely paralyzed post-stroke patients or individuals with tetraparesis, (iii) the focus of BMI-systems’ ability on detecting intention (i.e., the neurophysiological signals changes classification accuracy). Only a few studies involving patients associated motor-behavioral and functional changes to brain activity changes (24).

During a BMI-based motor-training, increase and control of targeted brain activity is the first important step to achieve the intended goal (10, 11, 26). This is accomplished through feedback mediated learning. The feedback is used to successfully complete specific tasks and to provide reward, even if feedback output devices are frequently controlled in a binary mode only, i.e., opening and closing the hand. Often these BMIs imply a delayed proprioceptive feedback in the intention-action loop, especially when motor-imagery (MI) and an external device are combined and applied to severe cases, such as a typical MI cursor BMI-task whose successful completion triggers a robotic action (10, 11) or FES stimulation (26). Four exceptions were presented by Ramos-Murguialday et al. (21, 23, 24) and Gomez-Rodriguez et al. (22), who reported an on-line intention decoding example during a BMI-task to flex or extend the hand and forearm with a robotic device. These on-line applications provided participants with a non-delayed haptic feedback during the task (not at the end of the mental task) allowing contingent stimulation of involved afferent and efferent neural networks; however, these studies involved mainly healthy participants, except for two stroke patients in Gomez-Rodriguez et al. (22), who were not clinically described, and the two patients groups described in Ramos-Murguialday et al. (24). These first studies employing non-delayed feedback (i.e., closed-loop paradigm and on-line feedback) emphasized the advantages to closely relate the feedback with intended actions or goals, and consequently to promote cortical reorganization (4, 28). Moreover one study has proven large motor-function improvements in presence of a moderate BMI-performance (27). Therefore, BMI-based motor-training could offer further explanations of the role of feedback into the mechanisms underlying recovery of motor-functions (3, 4, 29).

In this study we describe a new closed-loop BMI-application with assisted-feedback of force in which the electroencephalographic (EEG) oscillations of sensori-motor rhythms (SMR) were used to continuously assist the intended movement by adding a force to the actual motor execution. This added force is proportional to the contralateral rolandic rhythm de-synchronization caused by movement intention and movement execution (ME). We call this BMI-system an “assisted-force-feedback” BMI (29). The main elements of this application are: the use of a motor execution task (not a MI task), the type of feedback and the concomitant recording of motor kinematic performance (8, 30). In particular and differently from other methods, the proposed augmented feedback allows a fast (non-delayed) contingent and proportionally modulated (non-binary) activation of afferent proprioceptive and motor output neural pathways for motor rehabilitation in an operant learning context.

To demonstrate the feasibility, a patient with a moderate impairment of the upper limb motor-function, due to hemiparesis, was trained for 2 weeks with pre- and post-assessment evaluations. In accordance with the above considerations we report the methodology, neurophysiological signal changes, behavioral outcome during training and motor-task kinematic outcome.

## Materials and Methods

### Patient

A 25-years-old moderate right-hemiplegic female chronic stroke survivor participated in the study. She suffered from a left-sided thalamic-capsular bleeding at the age of 16, due to a vascular malformation. A surgical intervention at the age of 17 stabilized her conditions. After 6 years, a right-hemiplegia with tingling and numbness due to the peri-lesional edema, probably caused by the intervention, occurred. She was admitted to the S.Camillo Hospital, for a short hospitalization period, where she underwent the BMI-training (see BMI Protocol) with both arms. Her disability was characterized by a limited right-arm and forearm control, right-hand grasping, external rotation, and a restricted volitional movement of the fingers (spasticity). However, she was able to use her right-hand or arm for some daily living activities (i.e., open a door, objects grasping), and was able to walk slowly without external aid. The chronic character of her impairment was assessed by multiple functional measurements after the surgical intervention (6, 3, and 1 months before inclusion in the present study). The following clinical tests were assessed 2 days before the experimental protocol: Fugl-Meyer Assessment for Upper Extremity (FMA-UE, score 40/66), sensibility (S, score 21/24), Modified Ashworth Score (MAS, score 4, biceps brachii, pectoralis major, flexor carpi, flexor digitorum profundus, flexor digitorum superficialis were measured), Reaching Score (RS, score 21/36), and Nine Hole Peg Test (NHPT, score 5 p./50 s).

The standardized language test (Aachener Aphasie Test, AAT; token test score: eight minimal deficit; comprehension score: seven slight deficit) and cognitive assessment was carried out to ensure understanding capability and execution of the proposed task. The participant was recruited for the study because she was able to initiate and complete the target-reaching task of our protocol, albeit with limited performance (inclusion criteria). The unaffected arm was used as control since neither placebo control was possible nor different control conditions. The experimental protocol was approved by the Ethical Committee of the S.Camillo Hospital. Written informed consent was obtained from the participant according to the Declaration of Helsinki.

### BMI motor-task

The BMI-application was designed to perform robot-aided upper limb tasks exploiting an operant learning paradigm (4, 29). It relies on two basic principles (see Control of the Robotic Device):

- to provide brain-signal-based continuous proportional modulation of assisted-force-feedback (closed-loop);
- to estimate motor-behavioral kinematic outcome.

**Figure 1:**
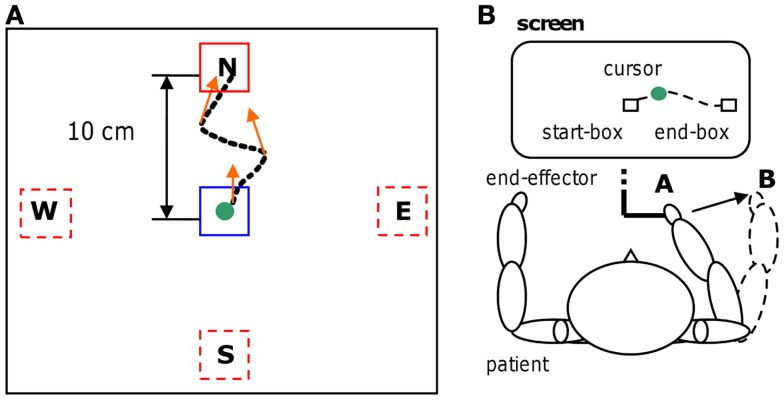
**Motor-training**. Four targets “center-out” task **(A)** with an example of a trajectory and some assisted-force-feedback vectors (orange arrows); physical representation **(B)** of the reaching task. The trajectory is shown for clarity, but during the motor task was never present.

**Figure 2:**
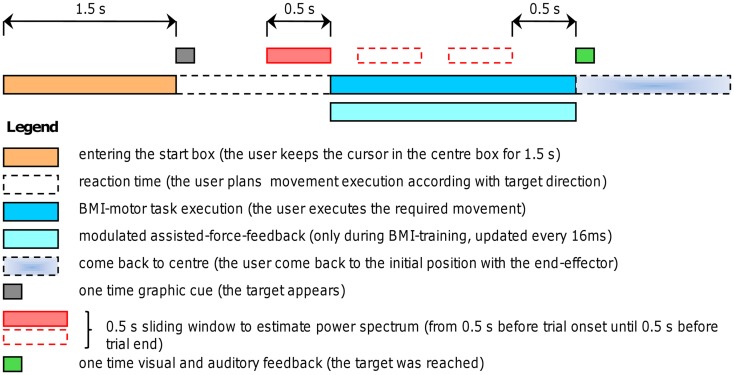
**Experimental procedure**. Timing of self-paced trials with and without assisted-force-feedback (the force applied to the robotic arm was continuously updated every 16 ms). A self-paced trials starts by entering the center box, after 1.5 s a target appears (one out of four possible directions), thus the user plans the movement execution and initiates the movement toward the target; as soon as the target was reached (i.e., at the end of the trial) both visual and auditory feedback inform the user about the task completion; then the user comes back to the center position to start another trial. During BMI-training an assisted-force-feedback was provided according to the algorithm explained in Section “On-Line BMI Assisted-Force-Feedback.”

A robotic device (PHANTOM Premium 3.0/6DOF, Sensable Technologies) was used to supply assisted-force-feedback proportional to event-related de-synchronization which was obtained using the BCI2000 platform [(31); www.bci2000.org]. Since the exercises required a volitional motor output we used oscillatory brain activity changes denoted here as movement-related de-synchronization (MRD). The EEG was recorded using 16 Ag/AgCl scalp electrodes located over fronto-central, central, and centro-parietal areas, according to the International 10–20 System (Fz, Cz, Fc1, Fc2, F3, F4, Fc5, Fc6, C3, C4, Cp1, Cp2, Cp5, Cp6, P3, P4), with a sampling rate of 512 Hz, and two pre-conditioning filters: a band-pass from 0.1 up to 60 Hz and a 50 Hz notch (gUSBAmp, g.tec GmbH). The robotic device allowed recording kinematic parameters of the movement (position and speed) every 1 ms. The experimental protocol consisted of a series of standard target-reaching tasks over a horizontal plane. The patient was seated comfortably in front of a table covered with a slippery surface and was asked to hold the robotic device stylus (i.e., the end-effector) and to focus on the computer screen. The position of the end-effector was always displayed as a cursor (diameter correspondent to 9 mm, cursor’s trajectories were never displayed) on the screen. During the repetitive task, the patient grasped the stylus device and controlled the position of the cursor on the screen receiving visuospatial (watching the cursor) and proprioceptive muscular force (contracting and moving the muscles) feedback. The patient underwent self-paced four targets “center-out” task: one out of four (N, E, S, and W) target positions was randomly selected in each trial for the ME (see Figure 1). Each target was represented by a white square of 18 mm. After an anticipation period of 1.5 s with the cursor in the center box (18 mm) a target appeared. The patient was asked to move the cursor, controlling the end-effector from the center to the target in a pre-defined time window (0.5÷0.7 s) and to perform the task as accurate as possible (see Figures 1 and 2). This interval was chosen on the basis of Fitts’ law, reviewed for the two-dimensional tasks by Scott MacKenzie and Buxton (32). A coherent visual and auditory feedback was provided at the end of each movement depending on its duration: the target exploded with a sound, if the duration of movement was between 0.5 and 0.7 s (i.e., “correct” trial); the target became red if the duration was below 0.5 s or it became blue if the duration was longer than 0.7 s. As soon as the target was reached, the patient was asked to move back to the center box and to repeat the task. The center-target distance was 10 cm (visual angle equal to 8°). A single run comprised 80 movements (20 trials for each direction). Before starting each run, a 40 s rest period was recorded while patients’ arms rested on the table. Triggers were saved on EEG traces to distinguish *rest* and *movement* conditions. A session consisted of three runs. During training on-line assisted-force-feedback was provided in each trial depending on brain activity changes as explained below (see On-line BMI Assisted-Force-Feedback). Summarizing, during training the patient received also a modulated assisted-force-feedback perceived haptically as a force supporting ME.

### BMI protocol

**Figure 3:**
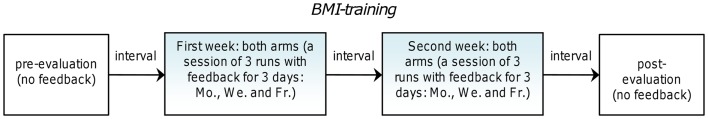
**Experimental protocol**. Interval between blocks consisted of 2 days.

The robot training was performed on both healthy and paretic arms in daily sessions during 2 weeks, 3 days a week, resulting in 18 runs for each arm (see Figure 3). Overall treatment duration was adapted to the hospitalization interval of the examined patient. A pre-evaluation session without assisted-force-feedback was performed 2 days before BMI-training on both arms of the patient as baseline assessment. An identical post-evaluation session was carried out without assisted-force-feedback as final assessment 2 days after the last BMI-training session. The “center-out” reaching task was employed during the robot training as well as in pre- and post-evaluations. A similar study (19) which exploited the same robot assistive device used here, but in an open-loop modality, reported results difficult to compare because of different types of exercise and measured outcome. For this reason we chose to use the unaffected arm of the same patient as control condition.

### EEG off-line data processing

Electroencephalographic fluctuations of amplitude greater than 100 μV caused by electrodes displacement or motion were marked and excluded from successive analysis. Direct current offset adjustment and common average reference (CAR) filtering were carried out to reduce low-frequency components in the spectral analysis. Successively, data were segmented in two classes: *rest* and *movement*. Each detected valid interval was used to estimate the power spectrum using the maximum entropy method (MEM), implemented in the BCI2000 platform and having a window size of 0.5 s, window step 4 samples and model order 16. The length of the analysis window (0.5 s) and overlap were chosen mainly on the basis of previous studies (4, 11, 21, 22) as it represented a good trade-off between the accuracy of power spectral estimates and the typical trial duration of the proposed motor-task (about 600 ms, see Kinematic Outcome, Table 1). The MEM analysis window started 0.5 s before a *movement* interval onset, continued through this interval as a sliding window with steps of four samples to replicate the on-line settings (see Figure 2), and ended 0.5 s before a valid interval offset. All power spectrum estimates within the same valid interval were averaged. In this way we estimated, in each trial, both the initial de-synchronization, during movement planning (i.e., intention to move), and the movement-related de-synchronization during trial execution. For each recorded channel the frequency analysis provided spectral power estimates subdivided in equally spaced bins starting from 0.5 up to 30.5 Hz with a bin width of 3 Hz. Spectral features distributions of the two conditions were then compared using the highest explained variance (*R*^2^ values) as reported in some studies (10, 21, 23, 26). These *R*^2^ values were successively used as criteria to select electrodes and frequency bands to provide feedback (see Feedback Sources Selection).

**Table 1:** **“Correct” trials kinematic outcome**.

Categories	Description	Healthy arm			Affected arm		
		Pre-evaluation (*N* = 134) Mean (STD)	Post-evaluation (*N* = 201) Mean (STD)	Statistics	Pre-evaluation (*N* = 93) Mean (STD)	Post-evaluation (*N* = 122) Mean (STD)	Statistics
				*p*-Value			*p*-Value
General behavior	% Of “correct” trials	55.8 (16.3)	83.8 (6.5)	0.046*^a^	38.8 (14.2)	50.8 (8.3)	0.184^a^
	Reaction time (ms)	380 (49)	417 (47)	<0.001**	487 (460)	528 (75)	<0.001**
Raw arm control	Duration (ms)	604 (52)	604 (50)	0.959	590 (52)	610 (49)	0.008**
	Mean speed (mm/s)	159 (13)	157 (15)	0.331	161 (14)	155 (14)	0.005**
Fine-tuned distal arm control	Speed peak (mm/s)	275 (33)	226 (23)	<0.001**	271 (40)	234 (29)	<0.001**
	Orthog. error (mm)	5.2 (2.3)	4.5 (1.8)	0.003**	6.9 (3.4)	4.7 (2.2)	<0.001**
	Area error (mm^2^)	15.1 (15.6)	11.0 (11.9)	0.021*	22.1 (28.1)	10.8 (12.2)	<0.001**

Statistics were performed by means of Wilcoxon test, except for the percentage of “correct” trials for which we used the Kruskal–Wallis test ^a^. Significance level: *p < 0.05, **p < 0.01.

A cross-check method was used to verify spectral power decrease during a *movement* condition in pre- and post-evaluations. For each valid trial of a run we estimated the movement-related de-synchronization of each electrode used for feedback as the fraction of spectral power decrease during a *movement* condition interval in comparison to the mean spectral power of the *rest* condition of the same run. The following formula was used for each valid *movement* condition trial (and for each selected electrode) of a single run: *P_n_* = [*P_i_*−*P_r-_*_mean_]/*P_r_*_-mean_ (where *P_i_* is the power spectrum value of each valid *i-th* trial in a run and *P_r-_*_mean_ is the *rest* condition mean spectral power of the same run). The main difference with respect to the *R*^2^ values estimation relies on the fact that power spectral values were normalized considering each single run separately. This normalization allowed a comparison of the movement-related de-synchronization across runs and specifically between pre- and post-evaluations.

### Feedback sources selection

Consistent with previous studies’ findings on event-related de-synchronization and motor tasks (33, 34), we selected electrode locations and frequency bins more related to a motor execution task than to a MI task. Therefore we: (i) preferred regions where cortical activities were correlated with the requested motor-task and its proprioception [proprioceptive afferent inputs could enhance the possibility to distinguish the movement condition; (21–23)], (ii) selected electrodes and frequency bins yielding highest and significant explained variance (*R*^2^) between *rest* and *movement* conditions of pre-evaluation sessions performed by the same patient before the training [without assisted-force-feedback; (21, 23, 24, 26)], (iii) chose frequency bins closely related to SMR activity and to avoid electromyographic artifacts (33, 34), (iv) used contralateral EEG electrodes, to constrain selection on ipsilesional brain areas (for the paretic arm) and contralesional brain areas for the left-healthy arm (to ensure almost equal control conditions for arms comparison). Therefore, we chose for the BMI-feedback electrodes over the primary motor cortex (M1), primary somatosensory cortex, and secondary somatosensory cortex. For the right-affected arm we used EEG power at 14–17 Hz and electrode locations C3, Cp1, P3, Cp5, while for left-healthy arm we used EEG power at 11–14 Hz and electrode locations C4, Cp2, P4, Cp6. This configuration remained unchanged during on-line feedback BMI-training (see Figure 3).

### On-line BMI assisted-force-feedback

For each arm power spectral components of selected frequency bins and channels were used as input of the standard linear classifier implemented by BCI2000 platform. Hence, the specified spectral components were linearly combined with equal weights of −1. The result was normalized (zero mean, unit variance, a standard operation computed by BCI2000 software) with respect to the *rest* period of each training run to adaptively take into account changes in the shape of *rest* period power spectral distribution across runs (21, 23, 26). We defined this normalized result as the *neuro-feedback* (*NFB*) value. A power spectrum decrease of selected locations and frequencies was reflected by positive values of *NFB* (since we used negative weights for the classifier). The *NFB* value was updated on-line every four samples (see EEG Off-Line Data Processing) as soon as the patient started the movement alone and for the total duration of task execution. In this way a continuous updating of the force to be applied to the robotic arm (to aid patient movement) was possible. If a positive *NFB* value was detected at movement onset, this can be attributed to movement planning phase (i.e., intention to move toward the target), because the analysis window used to compute the *NFB* value started 0.5 s before this time-instant; successive *NFB* values referred to either intention to move and/or ME. When the target was reached and during the (back-) path to the center box the assisted-force-feedback was disabled. Using pre-evaluation data of the patient, we separated *rest* and *movement* spectral power distributions optimizing a threshold *T* (equal to 0.5 for both arms) in terms of *rest* condition distribution standard errors (i.e., Standard Deviation). The threshold value *T* was used to compute the assisted-force-feedback factor as follows (i.e., the coefficient used to compute the assisting force module, denoted as *AFF*): *AFF* = *NFB-T*. This operation ensured the activation of the assisting force when at least a minimum necessary movement-related de-synchronization occurred (i.e., a deviation of at least 0.5 × STD of the de-synchronization from the mean of the power spectrum distribution at rest). The assisting force factor was used during a trial to compute the force assistance with the following rules: (i) no force in absence of selected frequency bands and electrodes power decrease (*AFF* ≤ 0); (ii) a force proportional to the movement-related de-synchronization, using the assisting force factor as linear coefficient (*AFF* > 0), always directed to the target (target directed force: *TDF* = *AFF*⋅*C*, were the constant *C* was separately determined for the two arms, see Control of the Robotic Device); (iii) the force module was limited to the maximum force constraint of 10 N, independently of further assisting force factor increase; (iv) the force module was continuously updated during a trial every 16 ms (*SampleBlockSize* = 4 combined with a 1000 Hz robotic device servo-loop rate; every two sample blocks the assisting force factor was updated). The target directed assisting force was applied to the end-effector of the robotic device. Summarizing, an additional force always directed to the target was provided during training runs every time the patient produced a relevant movement-related de-synchronization intending and executing the movement (3), thus exploiting a smooth and modulated assistive-strategy (14).

### Control of the robotic device

Phantom device and graphics interface were controlled by an external program connected to BCI2000 by an UDP network protocol. Communication with BCI2000 platform is bi-directional. This external program, referred as “robotic interface,” is characterized by two concurrent processes: the first one controls the robotic arm and performs kinematic measurements (servo-loop rate equal to 1000 Hz); the second one handles the graphic interface adjusting the cursor position and scheduling each trial phase according to the timing of the task (see Figure 2). During an on-line session the robotic interface sends the task execution status (*rest* condition, target appearing, trial start, and target reached) to BCI2000, while BCI2000 transfers the *NFB* value to the robotic interface every sample block. The latter drives the robotic arm updating the force to be supplied to the end-effector. This is accomplished calculating the assisted-force-feedback factor according to the previously explained algorithm (see On-Line BMI Assisted-Force-Feedback) and determining the direction of the force depending on the actual cursor position. For the two arms we used two different constant factors *C* to evaluate the module of the assisting force based on mean maximum movement-related de-synchronization estimated through pre-evaluation runs data (left-healthy arm: *C_h_* = 7.0 N; right-affected arm: *C_a_* = 3.8 N). Crisp changes of force feedback were avoided by adjusting internal robotic device parameters. During a run, the end-effector trajectory and instantaneous speed time-courses of each trial were recorded. Trial duration and mean speed mainly refers to the raw arm control for which the patient received the mixed visual and auditory feedback at the end of a trial. The ME accuracy is estimated by means of two trajectory-related parameters: orthogonal error and area error (fine-tuned distal arm control, for which the patient did not receive any feedback). These two measures refer to the trajectory error using as an ideal path the (invisible) segment that joins center box and target box: orthogonal error is the maximum orthogonal trajectory displacement with respect to the ideal path; area error is the measure of the area comprised between real trajectory and ideal path. These data allowed a comprehensive description of the kinematic performance as explained in the Section “Kinematic Outcome.”

### Statistical analysis

The statistical analysis compared conditions (*rest* and *movement*) and pre- and post-evaluation estimates and outcomes. Wilcoxon rank-sum test was applied for trials comparison. To compare the percentage of successfully completed trials within a run (i.e., “correct” trials) we employed the Kruskal–Wallis test since assumption criteria to apply more advanced statistics were violated (we had only one value per run resulting in three “correct” trials percentage values for both pre- and post-evaluations). Feedback and kinematic relationship during training were assessed by Spearman correlation.

## Results

### Neurophysiological data

**Figure 4:**
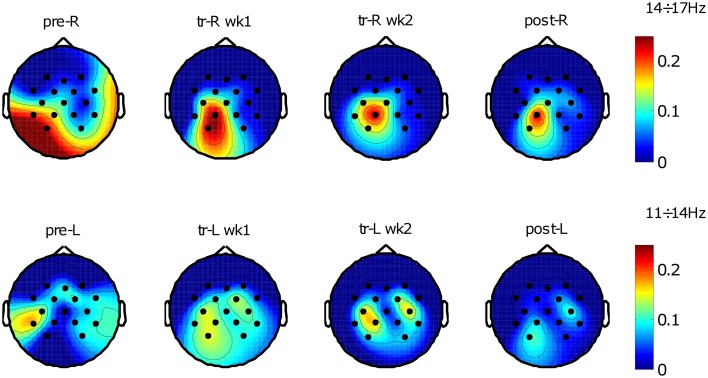
**Brain oscillations before and after training**. *R*^2^ maps of the two conditions across training (*rest* and *movement*); “pre” refers to pre-evaluation, “tr” refers to training, “post” refers to post-evaluation, “wk” refers to week (1 or 2), “R” refers to the right-hemiplegic arm, and “L” refers to the left-healthy arm. *R*^2^ values were evaluated between the two conditions with a variable number of trials depending on protocol phase and trials rejection percentage. Top *R*^2^ maps show spatial distribution related to the frequency bin 14÷17 Hz identified for the right-affected arm using pre-evaluation data (number of trials *N*: pre-R, *N* = *221*; tr-R wk1, *N* = *659*; tr-R wk2, *N* = *501*; post-R, *N* = *215*). Bottom *R*^2^ maps show spatial distribution related to the frequency bin 11÷14 Hz identified for the left-unaffected arm using pre-evaluation data (number of trials *N*: pre-L, *N* = *216*; tr-L wk1, *N* = *662*; tr-L wk2, *N* = *515*; post-L, *N* = *230*).

Selected frequency bands power spectral components of *rest* and *movement* conditions were compared by their explained variance *R*^2^ values across electrodes, which resulted in a series of maps describing the topographic evolution of the relevant cortical activities starting from pre-evaluation assessment to final assessment (see Figure 4).

**Figure 5:**
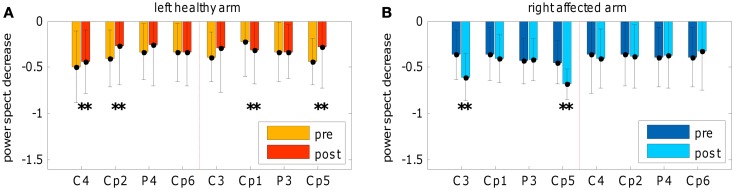
**Spectral power decrease**. Comparison of spectral power decrease during *movement* execution between pre- and post-evaluations. **(A)** Frequency bin from 11 to 14 Hz for left-healthy arm and **(B)** frequency bin from 14 to 17 Hz for the right-affected arm. The graphs depict the movement-related de-synchronization as the fraction of spectral power decrease during a *movement* condition interval in comparison to the mean spectral power of the *rest* condition (of the same run); for each electrode mean and standard deviation was represented. The normalization to the mean spectral power of the *rest* condition allows a comparison between pre- and post-evaluations by means of Wilcoxon test (for the number of pre- and post-evaluations trials see Figure 4 description). Electrodes showing the largest effects are slightly different than those showed in Figure 4 because the normalized computation is different from *R*^2^ estimation (see the cross-check method explained in Section “EEG Off-Line Data Processing”). In addition, significant changes of power decrease referred to the comparison between pre- and post-evaluations.

The topographic evolution consists of a focalization (i.e., reduction of the extent) of the brain areas that discriminate the two conditions. This process occurred during the treatment and was confirmed in the post-sessions by a significant SMR de-synchronization difference between pre- and post-evaluation of both hemispheres (see Figure 5). For the affected arm, the focus of increased de-synchronization was mainly located in the ipsilesional hemisphere (see Figure 5B), while at the healthy hemisphere a significant decrease of the de-synchronization occurred (see Figure 5A), probably reflecting a pre-post habituation effect.

**Figure 6:**
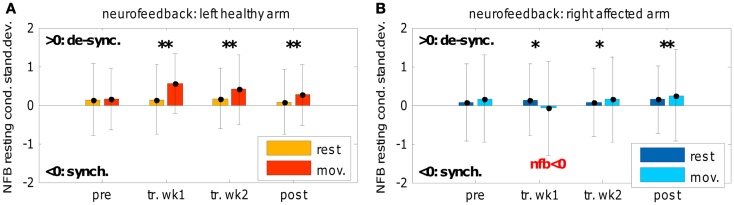
**Neuro-feedback**. *Neuro-feedback* evolution across training and in pre- and post-evaluations: **(A)** left-healthy arm and **(B)** right-affected arm. The *neuro-feedback* was obtained linearly combining power spectral components of selected electrodes and normalizing (zero mean, unit variance) with respect to the *rest* period of each training run; *y*-axis is represented in terms of *rest* condition standard errors (off-line corrected, without artifacts). Positive values of *neuro-feedback* reflected a movement-related de-synchronization; “pre” refers to pre-evaluation, “tr” refers to training, “post” refers to post-evaluation, “wk” refers to week (1 or 2). Wilcoxon test was applied between *rest* and *movement* conditions only.

Due to the classification algorithm (i.e., negative weights) and variance-normalization we expected for the *NFB* variable a mean value around zero at *rest* and a positive value during the *movements* (reflecting a movement-related de-synchronization). Figure 6 confirms this expectation with regard to the healthy arm: a significant distinction between *movement* and *rest* conditions during the training and the post-session can be noted. Differently, for the affected arm a significant movement-related de-synchronization can be observed in the second week of the training only, as well as in the post-session; while during the first week of training this neurophysiological change was not visible since we obtained on average a negative *NFB* value (i.e., synchronization).

### Behavioral outcome during BMI-training

Consistently with the required motor-task we focused the attention on correct trials percentage and the presence of the assisted-force feedback during training. Considering the healthy arm, the patient received an assisted-force-feedback in 67.4% of training trials only, with a mean force of 1.64 ± 1.49 N (mean force peak of 2.68 ± 2.24 N); in 72.7% of the total training trials she successfully completed the task reaching the target between 0.5 and 0.7 s; in remaining trials she was too fast or too slow. During training of the healthy arm there were no significant relationship between the number of correct trials and the presence of the feedback (no. of correct trials vs. no. of all trials with feedback: *r* = −0.33, *p* = 0.181; no. of correct trials vs. no. of only correct trials with feedback: *r* = −0.01, *p* = 0.968); similarly, the mean force peak and the number of only correct trials with feedback showed a non-significant correlation (*r* = 0.12; *p* = 0.644). With regard to the affected arm, the patient received an assisted-force-feedback in 58.1% of training trials only, with a mean force of 0.88 ± 0.9 N (mean force peak of 1.57 ± 1.24 N); only in 35.6% of the total training trials she successfully completed the task. Differently from the healthy arm, during training of the affected arm there were significant relationship between the number of correct trials and the presence of the feedback (no. of correct trials vs. no. of all trials with feedback: *r* = 0.66; *p* < 0.01; no. of correct trials vs. no. of only correct trials with feedback: *r* = 0.75; *p* < 0.001); in line with these results, the mean force peak and the number of only correct trials with feedback showed a significant correlation (*r* = 0.73; *p* < 0.001).

### Kinematic outcome

We separated kinematic results, obtained by the comparison between pre- and post-evaluations, in three categories to highlight physiological differences: general behavior, raw arm control (mainly related to trunk and proximal muscles to approach the target), and fine-tuned distal arm control (roughly related to distal muscles to reach the target with a straight line). Percentage of “correct” trials is given in Table 1. Furthermore, the following measures were summarized taking into account the portion of the trials successfully completed only: reaction time (general behavior); duration, mean speed (raw arm control); speed peak, orthogonal error, and area error (fine-tuned distal arm control).

For the left-healthy arm, only changes related to fine-tuned distal arm control (i.e., spatial accuracy) showed a significant improvement, while reaction time appeared significantly slower in the post-session. Conversely, regarding the right-affected arm we observed a generalized improvement including the spatial accuracy. As for the healthy arm, reaction time was slower in the post-evaluation. The increase of reaction time should be examined together with the other kinematic results (see Discussion). Kinematic and behavioral effects resulted in an enhanced accuracy of the ME.

Task-related spatial accuracy was compared between arms by means of orthogonal error and area error changes normalizing post-session data with mean results of pre-evaluation data. Each post-session “correct” trial value, for both orthogonal error and area error variables, was normalized as a fraction [*V_n_* = (*V*_post_-*M*_pre_)/*M*_pre_, where *V*_post_ is the value of the considered variable in each “correct” post-session trial and *M*_pre_ is the mean value of the same variable of all “correct” pre-evaluation trials]. These quantities explained for each arm the fraction of spatial accuracy improvement after BMI-training. Then the average values of the two arms were compared. Affected arm task-related spatial accuracy improvement was significantly higher than unaffected arm (post-evaluation “correct” trials: *N*_healthy_ = 201, *N*_affected_ = 122; orthogonal error change fraction, *OE*: *OE*_healthy_ = −0.14, *OE*_affected_ = −0.32, *p* < 0.00001; area error change fraction, *AE*: *AE*_healthy_ = −0.27, *AE*_affected_ = −0.51, *p* < 0.00001).

**Figure 7:**
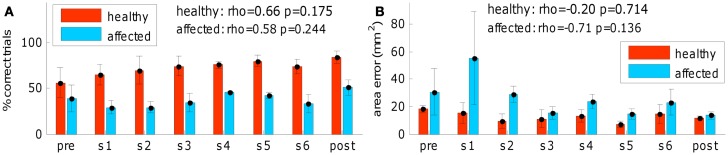
**Training process outcome**. **(A)** Successful task completion (general behavior) and **(B)** area error (fine-tuned distal arm control) across the training and in pre- and post-evaluations (red for left-healthy arm, light blue for right-affected arm). “pre” refers to pre-evaluation and “post” refers to post-evaluation. “s1,” “s2,” “s3,” “s4,” “s5,” and “s6” refer to session number (first week of training: “s1,” “s2,” and “s3”; second week of training: “s4,” “s5,” and “s6”). No sudden changes throughout the training sessions occurred. Spearman correlation of the two variables across the training sequence is reported for both arms.

Finally, a session-by-session analysis of the training process (see Figure 7) revealed that no sudden changes throughout the training sessions occurred, on the other hand a gradual improvement can be observed. Figure 7 shows, session-by-session, the trend of two main parameters across the training and in pre- and post-evaluations. The gradual enhancement is partially confirmed by the comparison (using Wilcoxon test) of most important kinematic parameters between the 2 weeks of training (% “correct” trials: *p*_healthy_ = 0.197, *p*_affected_ = 0.023; duration: *p*_healthy_ = 1.0, *p*_affected_ = 0.05; mean speed: *p*_healthy_ = 0.031, *p*_affected_ = 0.436; speed peak: *p*_healthy_ = 0.019, *p*_affected_ = 0.73; orthogonal error: *p*_healthy_ = 0.666, *p*_affected_ = 0.161; area error: *p*_healthy_ = 0.931, *p*_affected_ = 0.258).

## Discussion

In this study a new closed-loop BMI-application with assisted-force-feedback is described in a proof-of-principle prototypical demonstration. BMI-training was delivered to both arms of a patient with unilateral chronic brain injury for 2 weeks. This feedback was continuously modulated during a reaching movement task driven by rolandic rhythm oscillations. Neurophysiological and kinematic evidences reported here are strictly related to the repetition of the motor-task and the presence of the assisted-force-feedback: the patient was asked to move the cursor, controlling the end-effector from the center to the target in a pre-defined time window (0.5÷0.7 s) and to perform the task as accurate as possible. Since one single case is presented without control conditions, except the evaluation of the unaffected arm, the interpretation of the neurophysiological and kinematic evidences reported here is limited. Results are described as systematic observations only and the design of proper control conditions is discussed below.

Neurophysiological correlates showed an increase and focalization of the sensori-motor cortical activity used to control the BMI. A significant increase of selected SMR changes was found, particularly in the second part of the treatment and in the post-evaluation. In line with previous findings, these SMR changes reflect an increased de-synchronization during motor-training (11). The lack of an increased de-synchronization during right-affected arm exercises in the first week of the training could be explained with at least three factors: (i) motor-task execution may be too fast (i.e., about 600 ms) to induce a noticeable movement-related de-synchronization (most likely we observed only the onset of SMR de-synchronization); (ii) in some cases the assisted-force-feedback reached large values interfering with the assistive-strategy and distracting the patient during ME; (iii) threshold selection to provide feedback should be accurately selected in order to teach the patient more distinctively how to complete the task successfully. In addition, such neurophysiological patterns could be more specifically described using high density EEG recording. The focalization phenomenon was confirmed for movements executed with the right-affected arm in the post-evaluation (without assisted-force-feedback) and was associated with a significant increase of the SMR de-synchronization in two ipsilesional sites (C3 and Cp5) without significant effects on contralesional locations. Differently, we observed for movements executed with unaffected left arm a bilateral focalization. In the ipsilesional hemisphere two sites showed opposite behaviors: in Cp1 we observed an increased SMR de-synchronization, while in Cp5 we noted a decreased SMR de-synchronization. In the contralesional hemisphere a decreased SMR de-synchronization could be noted in C4 and Cp2 sites. The bilateral focalization seems to be related to the complexity of the task and involved brain areas: body spatial representation, target representation, visuomotor integration of target selection and movement selection (i.e., movement planning) and inter-hemispheric inhibition are parallel processes that involve parietal to frontal areas in both hemispheres during a reaching movement (35–38). However, the focalization suggests also successive reduction of resources and a progressive automation of the involved processes accompanied by sequential elimination of processing resources outside the sensory-motor areas.

The focalization covaried with improved kinematic spatial accuracy control. Analyzing behavioral and kinematic outcomes related to “correct” trials we observed a significant improvement of the fine-tuned distal control in both arms, mainly measured by spatial movement’s accuracy (i.e., orthogonal and area errors significantly decreased). Raw arm control showed a significant improvement in right-affected arm only; in particular, we observed a significant slowing. Behavior related to the motor-task was characterized by a significant increase of reaction time which appeared slower in the post-assessment for both arms. This effect is associated to a slightly prolonged movement planning phase, with enhanced accuracy of the ME (39, 40). Considering all these effects, during the BMI-training the patient improved her skill. As a consequence, in the post-evaluation we observed for the right-affected arm a prolonged movement planning (increased reaction time), slowing, a significant reduction of the spatial errors and an upward trend of “correct” trials. The first two effects (the extended movement planning and the slowing) allowed the patient to complete the task more often “correctly” than in pre-evaluation and more accurately. In addition, a direct comparison between arms shows that affected arm task-related spatial accuracy improvement was significantly higher than the unaffected arm. Although the gradual improvement on percentage of “correct” trials was non-significant for the affected arm, during the training a positive, strong, and significant relationship between this behavioral parameter and the assisting force provided to the patient was found, indicating the close association between *neuro-feedback* and behavioral performance. During the training of the unaffected arm a gradual improvement of the successful task completion was observed, but we did not find the same positive and significant relationship as in the affected arm. This could be explained by two inter-related factors: a higher mean and peak value of the force applied during training preventing correct movements and a ceiling effect on the number of “correct” trials due to the unaltered and precise motor-task execution.

The neurophysiological and motor-behavioral kinematic results might be associated with two fundamental determinants characterizing the BMI-training: the repetition of the task (with visual and acoustic feedback at the end of each trial) and the presence of the closed-loop assisted-force-feedback. Regardless of the positive evidence observed in this application, we are unable to separate these two factors and draw consistent conclusions about the effectiveness of this novel methodology. To this purpose proper control conditions should be designed. The main aim of such a control is to assess the effectiveness of this BMI-system in terms of motor-behavioral and functional recovery. As suggested by Ramos-Murguialday et al. (23), one possibility is to design a study where three groups are involved in the same training, each one receiving a different assisted-force-feedback contingency. The group A should receive a contingent feedback linked to SMR de-synchronization. The group B should receive a contingent feedback coupled with SMR-synchronization. While the third group should receive sham feedback [independent from brain activity (23, 24)]. A comparison of the three groups might supply evidence of the feedback modalities effectiveness, together with a systematic evaluation of the performance related to the duration of the training. In addition, follow-up measurements should be planned in order to verify whether learned motor skills are maintained over time (18). Another control condition could be a group of stroke patients trained with the same system in an open-loop modality with a pre-defined control of the force-field assistance and without brain activity involvement as reported in (19).

Another relevant aspect of the training design is the amount of the assisting force received by the patients because it strongly depends on their residual motor abilities. As the extent of motion increases patients should receive less assistance or even an opposing force. An exemplary case is reported by Fasoli et al. (18) where two types of goal-directed robotic therapies (assisted and progressive-resistive exercises) were delivered, without the BMI, to patients with different clinical conditions. Considering that motor abilities (i.e., functional recovery) can improve during a training protocol, future studies should take into account the overall duration (roughly short-term or long-term treatments). In a BMI context, this suggests that the amount and/or the sign of the robot assistance should be adapted to both the activation of the selected patterns to provide the feedback and to the duration of the training, or else, the motor abilities recovered by the patients. These observations can lead to different choices of the experimental setting (i.e., changing the sign of the proportional rule used to provide force assistance or to generate an opposing force), that in turn address and limit the study design, patients’ inclusion criteria, and definition of proper controls.

The combination of operant learning and the paradigm used for this experiment needs some residual motor abilities of the patients. This limits the applicability of this BMI-training to patients with a moderate motor impairment. The applicability might be extended to patients with severe disabilities changing the experimental design (i.e., the type of exercise, modality of gravity support, range of motion measurement, strength of the provided force assistance). In future studies cost-benefit ratio parameters in the evaluation of the proposed rehabilitation treatment and its effectiveness should be included. This encompasses a quantitative definition of the target population and a comparison with standardized and widely used rehabilitation interventions.

Finally, the presented BMI strategy approaches daily living movements more closely compared with delayed haptic feedback BMI-applications because ME is closely related to enhanced proprioception (41). This assisted-force-feedback BMI-application is designed for motor impaired patients with residual movements (29) and might open new possibilities in terms of robotic-rehabilitation.

## Conclusion

This study describes a BMI-based training with closed-loop continuous muscular assisted-force-feedback. Neurophysiological findings and kinematic/behavioral results, reported for a prototypical case, provide time-limited beneficial evidence for both the repetition of the task and the presence of the closed-loop assistance. This novel application might be useful to examine in depth neurophysiological phenomena and learning mechanisms underlying re-learning of specific motor behaviors. A proper controlled design is necessary to evaluate feedback modality effectiveness. Future robot-mediated rehabilitation protocols could be designed following this new learning strategy that allows to add sensory and proprioceptive information to the closed-loop motor-training.

## Conflict of Interest Statement

The authors declare that the research was conducted in the absence of any commercial or financial relationships that could be construed as a potential conflict of interest.
